# Two Faces of Greater Omentum

**DOI:** 10.1002/cph4.70073

**Published:** 2025-11-24

**Authors:** Czapiewska Monika, Mika Adriana, Abacjew‐Chmylko Anna

**Affiliations:** ^1^ Department of Pharmaceutical Biochemistry, Faculty of Pharmacy Medical University of Gdansk Gdansk Poland; ^2^ Department of Environmental Analytics, Faculty of Chemistry University of Gdansk Gdansk Poland; ^3^ Department of Obstetrics and Gynecology, Gynecological Oncology and Endocrinological Gynecology University Clinical Center Gdansk Poland; ^4^ Department of Gynecology, Obstetrics and Neonatology Medical University of Gdansk Gdansk Poland

## Abstract

The greater omentum, often described as a “plaster” of the abdominal cavity, exhibits remarkable regenerative and immunological properties. Its unique morphology—rich vasculature and a diverse cellular composition comprising adipocytes, endothelial cells, and leukocyte aggregates known as milky spots (MS)—facilitates immune surveillance, fluid uptake, and the secretion of neurotransmitters. Additionally, MS contribute to peritoneal immunity by capturing pathogens, promoting lymphocyte proliferation, and releasing cytokines and chemokines that recruit effector immune cells while limiting local inflammation. Structurally, this peritoneal extension shields visceral organs, prevents adhesions, and absorbs tumor secretions, yet paradoxically also provides a niche for metastatic spread. Moreover, the greater omentum is susceptible to various pathologies—vascular steal can deprive organs of blood, torsion and herniation threaten tissue viability, and ossification can transform the greater omentum into a rigid structure lacking protective properties. Notably, omentectomy has been associated with weakened antibacterial defense, underscoring its protective role. This review aims to explore the multifaceted nature of the greater omentum, emphasizing both its physiological benefits and the potential disadvantages associated with its alteration or removal.

## Graphical abstract

This review highlights the beneficial and negative effects of the greater omentum in the body: how the omentum contributes to healing and regeneration, but also how, in some conditions, it can promote inflammation and cancer spread.

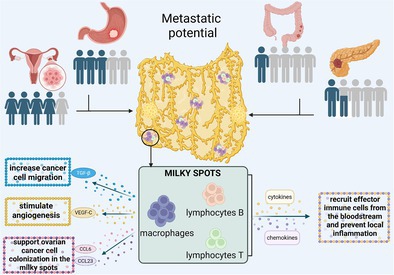

## 1 Introduction: What We Generally Know About the Omentum

The greater omentum serves several crucial functions within the human body, with its primary role being protective. Structurally resembling an apron, it is a two‐layer extension of the peritoneum—the largest and most complex serous membrane in the body—that envelops the visceral organs. The omentum provides mechanical protection to internal organs and prevents adhesions between the parietal and visceral peritoneum within the abdominal cavity (Bella et al. 2022).

**FIGURE 1:**
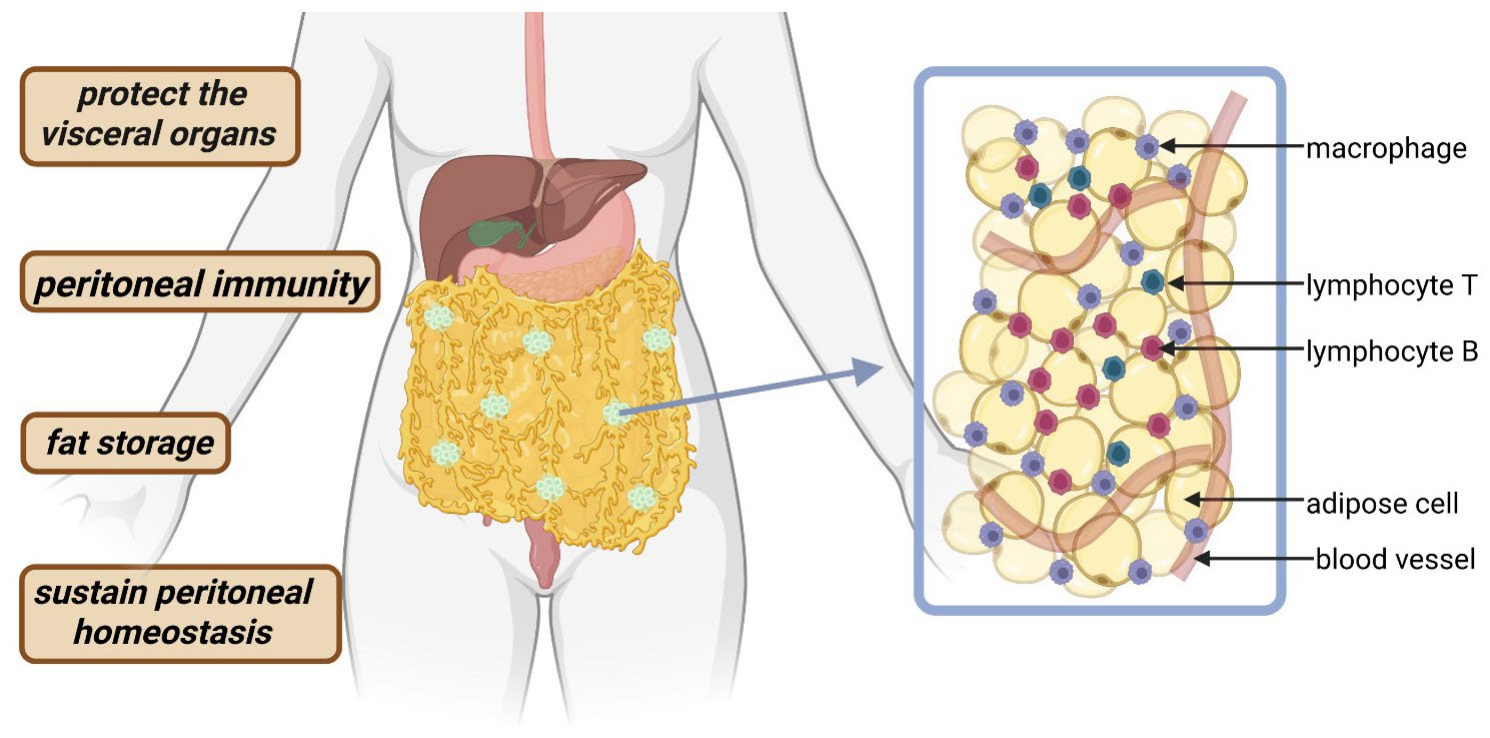
Anatomical location and functions of the omentum. Created by BioRender.com.

Anatomically, the omentum is situated in the anterior portion of the peritoneal cavity. It extends from the greater curvature of the stomach to the transverse colon, draping over the small bowel. Its surface area varies considerably, typically averaging around 500 cm^2^, with reported ranges from 300 to 1500 cm^2^ (Ghahremani 2023; Di Nicola 2019). Omental size correlates strongly with body weight, with increased fat deposition observed in obese individuals (Okabe 2024), which may influence metabolic and immune functions. The anatomical position of the omentum is presented in Figure 1.

The omentum exhibits unique immune capabilities, owing to its rich vasculature and unique cellular composition, including adipocytes, endothelial cells, and specialized leukocyte aggregates known as “milky spots” (MS). These MS are located in perivascular regions adjacent to the mesothelial cell layer, adjacent to adipocytes, and positioned near peritoneal perforations, known as stromata (Bella et al. 2022). Immune cells traverse these perforations, enabling interaction with peritoneal contents. Milky spots facilitate immune surveillance, fluid absorption, and the secretion of neurotransmitters such as dopamine, epinephrine, norepinephrine and choline acetyltransferase (Bahar and Rokkam 2024). They are pivotal in the maintenance of peritoneal immunity by scavenging pathogens and initiating inflammation and fibrosis (Meza‐Perez and Randall 2017).

In response to peritoneal insults, such as bacterial antigens, MS mediates lymphocyte proliferation secretes cytokines and chemokines that recruit effector immune cells from the bloodstream and prevents local inflammation (Gerber et al. 2006). Conversely, the surgical removal of the omentum has been shown to impair antibacterial defense mechanisms within the peritoneal cavity (Uzunköy et al. 2009).

Beyond its immune function, the omentum contributes to peritoneal homeostasis through mechanisms including fluid and solute transport, tissue and cell repair, angiogenesis, defense against infection and lipid storage, and acting as a reservoir for stem cells (Bahar and Rokkam 2024). Omental adipose tissue (OAT), primarily composed of fat cells, stores lipids such as neutral triglycerides, phospholipids, glycolipids, and gangliosides (Romanelli et al. 2019).

This review aims to explore the multifaceted nature of the greater omentum, emphasizing both its physiological advantages and the potential drawbacks associated with its alterations or removal.

## 2 Omentum—Cellular Composition

### 2.1 Milky Spots and Immune Function

Milky spots (MS) are small, specialized lymphoid structures within the greater omentum that play a critical role in immune surveillance and regulation in the peritoneal cavity as illustrated in Figure 1. MS possesses a distinct cellular composition, featuring a high amount of B lymphocytes and a unique cellular profile compared to other lymphoid organs (Figure 1). They also contain smaller populations of T lymphocytes and a relatively increased proportion of natural killer T cells (Meza‐Perez and Randall 2017). Notably, the greater omentum also hosts a unique population of regulatory T cells (Tregs), which, alongside NKT cells, produce high levels of interleukin‐10 (IL‐10), a cytokine crucial for modulating immune responses. These cell populations are significantly diminished in individuals with obesity (Lynch et al. 2009; Collins et al. 2009).

MS are formed during embryonic development. This process mainly occurs in the second half of fetal life, around weeks 20–35 of gestation, as primary lymphatic vessels differentiate and clusters of immune cells appear (Okabe 2024). Initially, these are small accumulations of macrophages, which over time develop into vascularised clusters and then into the actual milky spots. Macrophages are present at various stages of maturation and can easily enter or leave milky spots. These lymphoid microstructures are closely associated with the mesothelial layer covering the omentum, giving them an important role in local immune defense within the peritoneal cavity (Platell et al. 2000; Michailova and Usunoff 2004). The quantity of milky spots peaks in infancy and gradually decreases with age (Platell et al. 2000).

Histologically, MS appear as small, white lymphoid aggregates beneath the omental mesothelium and are not visible macroscopically (Di Nicola 2019). Hematoxylin staining, which colors MS blue, facilitates the assessment of their number, size, distribution, and topographical relationships (Schurink et al. 2019). MS develop either during fetal life or postnatally in response to peritoneal irritation or inflammation (Okabe 2024). Functionally, they act as immunologically, mediating visceral immune responses through antigen capture, particle clearance, and the regulation of lymphocyte trafficking (Di Nicola 2019; Pettet et al. 1956). They are sites of immune cell proliferation, differentiation, distribution and self‐renewal (Okabe 2024) (Liu et al. 2020). Despite their protective role, MS can also contribute to the peritoneal dissemination of cancer cells within the peritoneal cavity (Ng et al. 2025).

### 2.2 Omental Adipose Tissue and Lipid Metabolism

The greater omentum itself is a metabolically active adipose tissue that supports the intestines and abdominal organs, serving as a major depot of visceral white adipose tissue (WAT). Developed shortly after birth, it accounts for a substantial portion of total body fat (Chkourko Gusky et al. 2016). Approximately 97% of omental lipids consist of neutral glycerides, with smaller amounts of polar lipids such as phospholipids, glycolipids, and gangliosides (Romanelli et al. 2019). Purified omental lipids (POL) have demonstrated clinical utility in dermatology; they are employed as topical emollients and moisturizers, and have been shown to improve skin hydration, particularly in individuals with type 2 diabetes (Milani and Federici 2016). POL‐containing creams exhibit superior emolliency compared to glycerine‐based formulations (Puviani et al. 2022).

In obesity, omental adipose tissue serves as a reservoir for excess cholesterol and fatty acids (Jové et al. 2014). This accumulation reflects a failure of subcutaneous adipose tissue expansion, often due to genetic, ethnic, age‐related, or hormonal factors (Tchernof and Després 2013). When subcutaneous fat storage is inadequate, excess fatty acids are redirected to ectopic depots such as the omentum, epicardial adipose tissue, and visceral organs including the pancreas, heart, and liver. These lipids are stored as intracellular lipid droplets (Ralston et al. 2025).

In addition to fat storage, omental WAT plays an active role in tissue regeneration. It produces vascular endothelial growth factor (VEGF), a key mediator of angiogenesis and tissue neovascularization. This property has been harnessed experimentally, for instance, in promoting neovascularization in ischemic tissues of the brain, heart muscle, extremities and rabbit cornea (Goldsmith et al. 1984). Lipid profiling of the greater omentum provides insight into the functional diversity of its lipids and their contribution to immune modulation and tissue repair, through endocrine signals. Emerging evidence also suggests that cancer cells can stimulate lipolysis in omental adipocytes, transforming the omentum into a supportive niche for tumor growth (Chkourko Gusky et al. 2016).

The omentum, as a major reservoir of visceral adipose tissue (VAT), contributes significantly to the pathophysiology of metabolic syndrome. Compared with subcutaneous adipose tissue, VAT demonstrates reduced insulin sensitivity, higher rates of lipolysis, increased release of free fatty acids (FFAs), and elevated production of proinflammatory mediators such as interleukin‐6 (IL‐6) and C‐reactive protein (CRP). Because VAT drains directly into the liver via the portal circulation, hepatocytes are exposed to both an excess supply of FFAs from lipolytically active adipocytes and to inflammatory factors including tumor necrosis factor‐α (TNF‐α), IL‐6, interferon‐γ (IFNγ), leptin, and CRP (Fried et al. 1998; Trujillo and Scherer 2006). The influx of FFAs contributes to insulin resistance, enhanced hepatic glucose production, impaired insulin degradation, and subsequent hyperinsulinemia, which collectively promote triglyceride synthesis, hepatic steatosis, and the development of non‐alcoholic fatty liver disease (NAFLD). Additionally, FFAs promote nitroxidative stress and hepatic inflammation, favoring the transition from simple steatosis to non‐alcoholic steatohepatitis. Dysregulated adipokine production is another hallmark of metabolic syndrome, characterized by decreased secretion of insulin‐sensitizing and anti‐inflammatory adiponectin (Garciá‐Ruiz et al. 2018).

At the genomic level, studies using microarray approaches have identified distinct expression patterns in omental fat, including the down‐regulation of genes that normally activate lipolysis. Omental adipose tissue also has the capacity to increase circulating glucocorticoids, a phenomenon that predisposes individuals to obesity and insulin resistance and has been described as a form of “Cushing's disease of the omentum.” Furthermore, the omentum is an active source of adipokines such as leptin, RANTES, and resistin, which may help explain how intra‐abdominal obesity drives metabolic dysfunction. An additional mechanism involves the enhanced delivery of FFAs into the portal system, which directly impacts hepatic insulin handling and promotes insulin resistance. Altogether, the omentum appears to be a central regulator of metabolic dysregulation, linking visceral obesity to systemic metabolic disease (Collins et al. 2009).

## 3 Biological and Clinical Significance of Omentum/Omentum—A Natural Ally in Treatment

### 3.1 Regeneration Function

Regeneration involves complex biological processes that restore damaged cells, tissues or entire organs to their original functional state. Numerous studies have demonstrated the healing potential of the omental grafts in injured organs (Vernik and Singh 2007). The omentum's rich vascularization supplies oxygen and nutrients to graft sites, promoting cell survival and reducing the risk of necrosis (Di Nicola 2019). However, the precise mechanisms underlying its regenerative capacity remain incompletely understood (Di Nicola 2019; Shah et al. 2012).

Omental transposition—a surgical technique in which the omentum is mobilized to cover or envelop tissues that require healing—was first described in the late 19th century by Nicholas Senn, who used it to prevent leakage of intestinal anastomoses (Pettet et al. 1956). Subsequently, this approach has been applied to minimize gastrointestinal leakage (Liu et al. 2014; Bhat et al. 2006; Matsui et al. 2018; Tocchi et al. 2000) and reduce intra‐abdominal adhesions (Ariake et al. 2015).

Goldsmith et al. investigated the effects of omental transposition on the central nervous system. Their findings revealed enhanced cerebral blood flow and evidence of increased dendritic proliferation, increased synaptic connections and the development of new neural pathways. These effects are partly attributed to omental secretion of angiogenic factors, including vascular endothelial growth factor (VEGF) and fibroblast growth factor (FGF), which stimulate angiogenesis and are used in cerebral ischemia (Goldsmith et al. 1973). The omentum also possesses neurochemical activity. It contains the enzyme choline acetyltransferase, responsible for acetylcholine synthesis, the main component of cholinergic neurotransmission. Since acetylcholine levels are significantly reduced in the brains of patients with Alzheimer's disease, the omentum may enhance cholinergic signaling, although the mechanisms remain unclear (Goldsmith 2002; Li et al. 2024).

Additionally, the omentum contributes to wound healing and regenerative responses. Contact with damaged tissue activates the expansion of milky spots and enhances the secretion of growth factors, angiogenic mediators and progenitor cells that promote tissue repair (Wang et al. 2020). The omentum flap, a pliable and well‐vascularized tissue, is particularly effective for repairing extensive soft tissue defects (Liu et al. 2023).

In mice models with diabetes, where wound healing is often impaired due to poor glycemic control and vascular compromise, omental conditioned media have been shown to support angiogenesis, stimulate peripheral nerve regeneration at the wound margins, and promote collagen deposition in the early stages of wound healing (Li et al. 2023).

### 3.2 Reconstruction of Organs, Vessels and Nerves

The omental flap has been successfully applied in reconstructive surgery, including chest wall and mediastinal repair (Kreutz‐Rodrigues et al. 2023), as well as breast reconstruction (Devisetti et al. 2024; Ma et al. 2021). Pedicled omentum can also be used to reduce infection in cases of thoracic aortic grafts—a life‐threatening complication, the pedicled omentum flap provides a vascularized barrier that reduces infection risk and has been associated with successful recovery (Jamieson et al. 2012; Hernandez et al. 2020; Shah et al. 2013).

The omentum is also used in the management of lymphoedema, a chronic condition involving lymphatic fluid accumulation. In advanced cases, a vascularized omentum lymph node transfer is employed. Omental flaps, enriched with lymphatic vessels and nodes, can restore lymphatic drainage and substantially improve quality of life (Di Taranto et al. 2020; Goldsmith et al. 1967; Bordianu et al. 2024).

Moreover, the omentum's high angiogenic potential makes it a valuable adjunct in peripheral nerve regeneration. Adequate blood supply is essential for successful nerve repair, particularly in nerve transplants. Experimental studies in rats have demonstrated that the omentum accelerates axonal regrowth (Chamorro et al. 1993; Castañeda and Kinne 2002). Fay et al. (2021) reported that the omentum contains high levels of renal cell antigen‐1 (RECA‐1) and laminin, both crucial for axonal regeneration. In addition, the omentum secretes basic FGF, which promotes the proliferation of endogenous neural stem cells and supports neuroprotection (Ye et al. 2018).

### 3.3 Omentum‐Derived Bioink and Organ Repair

Recent advances in regenerative medicine have explored the use of omental tissue as bio‐ink for 3D printing of autologous patches. These patches can be transplanted to injury sites, to promote healing. In experimental models of chronic kidney disease, omentum‐derived patches alleviated tubular injury and downregulated genes associated with fibrosis (Jo et al. 2022).

The above paragraphs highlight the beneficial aspects of the greater omentum in maintaining physiological balance and supporting clinical applications. However, advantages represent only one side of the omentum's complex nature. There is evidence showing that the greater omentum can have a negative impact on the body. To fully understand its dualistic character, it is therefore necessary to present the unwanted pathophysiological conditions resulting from the existence of the greater omentum.

## 4 The Second Face of the Greater Omentum

### 4.1 Omentum as a Microenvironment of Tumor Migration and Site of Cancer Metastasis

Cancer metastasis is a dynamic and multistep process in which cancer cells disseminate from the primary tumor to distant sites, establishing secondary tumors. This step of cancer development complicates treatment and worsens prognosis. In fact, metastases are responsible for over 90% of cancer‐related deaths, and patients with metastatic disease exhibit markedly lower 5‐year survival rates compared to those with localized tumors (Castaneda et al. 2022).

Due to its anatomical proximity and absence of physical barriers, the greater omentum is a frequent site of metastasis for intra‐abdominal and pelvic malignancies, including ovarian, endometrial, gastric, pancreatic, and colorectal cancers (Feygenzon et al. 2017; Turan et al. 2014; Desai and Moustarah 2022).

### 4.2 Ovarian and Endometrial Cancer

Ovarian cancer among gynecological malignancies has one of the highest mortality rates largely due to its high metastatic potential and lack of effective early detection methods (Yeung et al. 2015). Consequently, most ovarian cancer cases are diagnosed after intraabdominal spread has occurred, primarily to the omentum. Up to 80% of high‐grade serous ovarian carcinomas metastasize to the omentum (Chehade et al. 2022; Bilbao et al. 2021).

Endometrial carcinoma (EC), the most common gynecological malignancy, is frequently diagnosed at an early stage and generally carries a favorable prognosis, largely due to the predominance of well‐differentiated endometroid histopathological subtypes. However, approximately 8% of patients with stage I disease already present with omental involvement, which upgrades them to stage III. Among EC subtypes, high‐grade serous carcinoma exhibits the greatest propensity for extrauterine dissemination—including omental, peritoneal, and lymphatic spread—occurring in 40%–50% of cases. Although serous EC represents only 5%–10% of all endometrial carcinomas, its clinical behavior closely mirrors that of high‐grade serous ovarian carcinoma, with similarly aggressive progression and poor prognosis (Joo et al. 2015).

### 4.3 Gastrointestinal Cancers and Omental Metastasis

The omentum is a frequent site of metastasis for gastrointestinal malignancies. In gastric cancer (SC), which is among the most common cancers worldwide, omental involvement is observed in approximately 53%–66% of cases (Wang et al. 2021). Pancreatic cancer (PC), characterized by its highly aggressive behavior and inherent resistance to chemotherapy, metastasizes to the omentum in about one‐third of patients (Feygenzon et al. 2017; Li et al. 2025). In colorectal carcinoma (CC), the third most prevalent malignancy globally, peritoneal metastases—including those to the omentum—are seen in up to 25% of patients, typically indicating advanced‐stage disease and correlating with a significantly poorer prognosis (Enblad et al. 2024; Pretzsch et al. 2019). It has been observed that, following tumor migration to the omentum, there are more milky spots present in the greater omentum (Krist et al. 1998). This most likely occurs through interactions between chemokines secreted by omental macrophages and receptors on cancer cells. Macrophages secrete CCL2 chemokines, which are recognized by CCR4 receptors on cancer cells and attract these cells to the milky spots (Krishnan et al. 2020). Conversely, a larger number of cancer cells promotes the influx of more immune cells and the formation of new milky spots. A high concentration of cancer and immune cells creates a compact “omental cake.” Immunological and metabolic functions, including the presence of blood vessels and immune cells, may facilitate tumor invasion, proliferation, and therapy resistance (Li et al. 2024).

### 4.4 Mechanisms of Omental Colonization

Cancer cells can reach the greater omentum via *direct intraperitoneal seeding*, *lymphatic spread*, or—rarely—*hematogenous dissemination*. Though vascular metastasis is uncommon, a murine parabiosis study showed that ovarian cancer cells could spread through the bloodstream with a predilection for omental colonization (Pradeep et al. 2014). A human study found that patients with hematogenous metastases often had concurrent omental involvement (Iwagoi et al. 2021).

Direct peritoneal seeding involves detaching tumor cells from the primary tumor and dissemination via the peritoneal fluid. This is exacerbated by lymphatic dysfunction, which leads to ascites accumulation—a hallmark of advanced disease and a factor contributing to poor prognosis (Di Nicola 2019).

Cancer cell attachment and subsequent tumor growth in the omentum are localized predominantly to *milky spots*—specialized immune aggregates that are highly vascularized and rich in immune cells. Metastatic tumor cells preferentially grow in sites rich in proangiogenic vessels which provide metastatic cells with the microenvironment conducive to survival and subsequent growth (Gerber et al. 2006). Macrophages in MS secrete proangiogenic factors such as VEGF‐C and TGF‐β. VEGF‐C stimulates angiogenesis and facilitates vasculature to the vasculature, while TGF‐β drives epithelial‐mesenchymal transition, enhancing cancer cell migration (Nowak and Klink 2020). Additionally, macrophages release chemokines CCL6 and CCL23, which support ovarian cancer cell colonization in the MS (Krishnan et al. 2020).

The omentum also affects tumor metabolism. It provides biochemical signals that enhance branched‐chain amino acid (BCAA) catabolism, which activates the mTOR signaling pathway, promoting cell cycle progression and ovarian cancer cell proliferation. These metabolic alterations are most evident in cancer cells located adjacent to the omentum, suggesting that omental secretions may modulate the metabolic behavior of nearby epithelial cells, particularly in fallopian tube‐origin carcinomas (Lusk et al. 2025).

### 4.5 Adipocytes and Immunosuppressive Microenvironment

The omentum is composed largely of adipose tissue, which supports tumor growth by supplying lipid energy sources and inflammatory cytokines. Omental adipocytes produce CCL2, IL‐6, and IL‐8, which recruit monocytes and promote their differentiation into tumor‐associated macrophages (TAMs) and myeloid‐derived suppressor cells (MDSCs)—key players in immune evasion. These cytokines also stimulate angiogenesis, invasion, and metastasis (Chkourko Gusky et al. 2016; Rezaeifard et al. 2014).

Furthermore, omental adipocytes activate pro‐survival signaling pathways in ovarian cancer cells, including p38 and STAT3 (Chkourko Gusky et al. 2016). Elevated CXCL‐10 cytokine levels have been found in patients with ovarian cancer compared to benign ovarian cysts and are also overexpressed in other malignancies such as colon and breast cancer (Kim et al. 2021; Song et al. 2021). IL‐10, another adipocyte‐derived cytokine, impairs antigen‐presenting cell function and inhibits pro‐inflammatory cytokines, weakening the antitumor immune response (Rezaeifard et al. 2014).

Gastrointestinal stromal tumors (GISTs), though usually arising in the stomach or small intestine, have occasionally been found in the greater omentum. Rare extra‐gastrointestinal stromal tumors (EGISTs) can also develop in the omentum, mesentery, or retroperitoneum. In addition, the omentum may serve as a metastatic site for melanoma and very rarely for primary neuroendocrine tumors (Toutounji et al. 2024; Yao et al. 2025; Taki et al. 2025; Chocarro‐Calvo et al. 2025).

### 4.6 Harmful Organism—Vascular Steal

Although the omentum is well‐known for promoting angiogenesis and vascularization, a “vascular steal” phenomenon has been observed. In patients undergoing esophagectomy measurements of tissue perfusion before and after surgery indicated that the greater omentum could divert blood flow away from the stomach, potentially compromising its postoperative perfusion (Lorenzo et al. 2023).

### 4.7 Omentum and Autoimmune Diseases

The omentum is also implicated in autoimmune diseases. In Crohn's disease, chronic inflammation in the gastrointestinal tract is accompanied by increased immune activation. The omentum contributes to this process by supplying dendritic cells that enhance local immune responses. In affected individuals, dendritic cells from the omentum show reduced expression of MHC class II molecules and a diminished capacity to activate the adaptive immune response. This immunological imbalance may contribute to excessive innate immune activity and the persistence of chronic inflammation (Schreiber et al. 1995; Bedford et al. 2006).

### 4.8 Remodeling Related Dysfunction of the Omentum in Disease

Omental torsion is a rare cause of acute abdominal pain and can be a torsion of the greater omentum. This is a condition in which the omentum twists in on itself and may resemble appendicitis or cholecystitis. The pathogenesis of omental torsion is not well understood. Primary omental torsion is thought to result from anatomical defects or vascular anomalies. Secondary omental torsion, which is far more common, is associated with pre‐existing abdominal pathologies such as tumors, injuries, scars, or hernias. Omental torsion may also occur as a consequence of passive displacement within the abdominal cavity, triggered by increased intra‐abdominal pressure due to heavy lifting, coughing, abdominal trauma, or excessive peristalsis (Ghosh and Arora 2014). The literature describes the case of a woman whose symptoms were suggestive of abdominal wall pathology. However, it turned out that the twisting of the omentum led to ischemia and necrosis and adhesion to the anterior abdominal wall (Sawada et al. 2022).

In turn, heterotopic ossification of the greater omentum is an abnormal formation of bone tissue within the greater omentum. The omentum undergoes extraskeletal calcification and bone tissue formation within its primary connective and fatty tissue (Çelik et al. 2019). This phenomenon is often preceded by inflammation or micro‐injuries that lead to fibroblastic proliferation. The process of ossification is linked to excessive activity of mesenchymal cells and fibroblasts, which can undergo phenotypic transformation into osteoblasts (Di Paolo et al. 2004). It is a rare but serious postoperative complication and can lead to small bowel obstruction, perforation of the abdomen and even death. To date, 29 cases have been described in the literature (Shi et al. 2011).

Further, hernias of the greater omentum account for approximately 1%–4% of all internal hernias (Chaouch et al. 2024). They cause intestinal loops to become trapped, in the greater omentum, which can lead to intestinal incarceration or strangulation. Hernias of the greater omentum may be congenital, associated with anatomical anomalies, or acquired, resulting from trauma, surgery, inflammation, or atrophy of the omentum (Pacheco et al. 2020). The main clinical symptom is acute abdominal pain, which mimics other conditions such as appendicitis. Early diagnosis and surgical intervention are crucial to prevent serious complications (Rudroff et al. 2013).

The publications presented in this review are a wide range of applications of the greater omentum—from immunology and oncology, through reconstructive surgery, to its use in the treatment of chronic diseases and tissue regeneration. An analysis of these reports enables identification of the main benefits of using omentum, such as its exceptional angiogenic properties, ability to modulate the immune response, regenerative potential, and the possibility of using it as a flexible surgical flap. At the same time, studies indicate limitations and risks, including complications following omentum harvest procedures, the possibility of adhesions, infectious complications and the phenomenon known as “vascular steal.” The table below summarizes the most frequently mentioned advantages and disadvantages of omentum applications (Table 1).

**TABLE 1:** Two faces of greater omentum.

Lp.	Feature of omentum	Advantages	Disadvantages
1.	Adipose tissue	Increase tissue vascularization by producing vascular endothelial growth factor and other angiogenic factors (Goldsmith et al. 1973)	High amount of lipids which enable the development, survival and invasiveness of tumors through direct lipid transfer (Chkourko Gusky et al. 2016)
			Activate pro‐survival pathways in cancer cells which leads to increased proliferation, migration and invasiveness of ovarian cancer cells (Chkourko Gusky et al. 2016)
			Secrets cytokines which promote cancer development (Chkourko Gusky et al. 2016; Rezaeifard et al. 2014; Kim et al. 2021; Song et al. 2021)
2.	Lymphatic vessels	The lymphatic vessels of the omentum can absorb excess fluid, proteins, immune cells, and other molecules from the intercellular space (Bahar and Rokkam 2024; Liu et al. 2020)	Lymph drainage disorders leading to swelling and inflammation (Di Taranto et al. 2020; Goldsmith et al. 1967)
3.	Milky spots	They create a local environment conducive to tissue repair and reconstruction (Litbarg et al. 2007)	
		Activation of the immune response and increase in the number of immune cells (Gerber et al. 2006)	
4.	Activate Omental flap	Prevent gastrointestinal leakage by physically sealing defects and actively supporting regeneration, angiogenesis and immunological control of infections (Liu et al. 2014; Bhat et al. 2006; Matsui et al. 2018; Tocchi et al. 2000)	
		Creating an environment conducive to axon regeneration and growth (Chamorro et al. 1993; Castañeda and Kinne 2002; Fay et al. 2021)	
5.	Omental Stromata		Microenvironment of gastrointestinal stromal tumors (Toutounji et al. 2024)
6.	Others	Omentum thanks to its unique extracellular matrix, rich in proteins, growth factors and bioactive ingredients can serve as Bio‐ink for 3D printing (Jo et al. 2022)	Omental defects, such as hernias or omental ossification (Shi et al. 2011; Chaouch et al. 2024)

## 5 Omentectomy—Indications for the Removal of the Omentum

In ovarian cancer surgery, omentectomy is a critical component of staging and cytoreduction. There are two principal types of omentectomy distinguished by the extent of tissue resection. Total omentectomy entails end bloc resection of the entire greater omentum, including its attachments from the greater curvature of the stomach to the transverse colon, often encompassing the gastrocolic, gastroepiploic, and splenocolic ligaments. This approach is indicated when there is extensive omental carcinomatosis or for comprehensive surgical staging. In contrast, partial omentectomy (also referred to as infra‐gastric or segmental omentectomy) involves resection of only the distal portion of the greater omentum, typically below the transverse colon, and is employed when disease involvement is localized or when preserving uninvolved tissue is preferred. The decision is guided by intraoperative findings, tumor distribution, and surgical goals for optimal cytoreduction. According to NCCN guidelines, omentectomy is routinely indicated during primary debulking surgery for epithelial ovarian cancer to ensure accurate staging and cytoreduction (NCCN 2025). Similarly, the ESGO–ESMO consensus recommends complete omentectomy for optimal surgical management in advanced‐stage ovarian cancer (Colombo et al. 2019).

In *endometrial carcinoma*, the role of omentectomy is more selective and histology dependent. While routine omentectomy is not recommended in low‐grade endometrioid histology due to the low risk of omental metastasis, it is considered *standard* in *high‐grade subtypes*, particularly *serous carcinoma*, *carcinosarcoma*, and *clear cell carcinoma*, due to their high propensity for peritoneal spread. In such cases, omentectomy is performed either as part of *surgical staging* or cytoreductive surgery in advanced disease, as serous endometrial carcinoma behaves biologically similar to high‐grade serous ovarian cancer (Concin et al. 2021). The European Society of Gynecological Oncology (ESGO) recommends omentectomy for staging in serous endometrial cancer and when gross disease is observed intraoperatively (Concin et al. 2021).

## 6 Post‐Omentectomy Effects

Beyond its established role in oncology—where omentectomy is performed either for cytoreduction of tumor mass or for staging to detect potential metastasis—relatively little is known about the broader health consequences of removing the omentum.

In the context of obesity and metabolic disorders, however, omentectomy appears to exert beneficial effects. Animal studies have shown that removal of the omentum reduces high‐fat diet–induced weight gain, thereby preventing the development of hyperglycemia, hypertriglyceridemia, insulin resistance, and non‐alcoholic fatty liver disease (NAFLD). Similarly, complete resection of the greater omentum has been associated with marked improvements in obesity and metabolic syndrome (Garciá‐Ruiz et al. 2018; Gabriely et al. 2002). Clinical studies support these findings, demonstrating that patients undergoing omentectomy in combination with adjustable gastric banding tend to experience greater weight loss compared to those receiving gastric banding alone. Moreover, omentectomy has been reported to improve oral glucose tolerance and insulin sensitivity while also lowering fasting plasma glucose and insulin concentrations sensitivity, and decreased fasting plasma glucose and insulin levels (Thörne et al. 2002).

The impact of omentectomy on postoperative infection and anastomotic healing remains a matter of debate. One clinical study reported that, in the setting of proctocolectomy with ileo‐anal anastomosis, removal of the omentum was associated with a higher risk of postoperative sepsis, including cases requiring reoperation (Ambroze et al. 1991). In contrast, other investigations have indicated that omentectomy does not impair the bactericidal activity of peritoneal fluid or increase bacterial counts in experimental models of cecal perforation (Thörne et al. 2002).

Evidence from rodent studies further suggests that omentectomy does not negatively influence the inflammatory phase of anastomotic healing. Instead, compensatory systemic mechanisms appear to support tissue repair, as peripheral leukocytes are recruited to the site of injury to sustain the healing process (Collins et al. 2009).

## 7 Conclusions and Perspectives

In summary, while the positive effects of omentum in treating diseases have been documented, it is still unclear whether there still may be negative effects in certain cases. The omentum appears to play a significant role in the regeneration process, as well as in the carcinogenesis and metastasis of tumors. The omentum in women has the most significantly impact in the development and spreading of ovarian and Fallopian tube cancers. Much of the research focuses on the functions and disorders of the greater omentum in women, with limited information available in men (below 1%); often limited to case reports related to stomach cancers. However, new reports are emerging that highlight the potentially harmful role of the omentum. Therefore, further studies are needed to explore the implications of both its beneficial and detrimental effects.

## Author Contributions

A.‐C.A. designed the study and put forward the conception. C.M. and M.A. conducted the literature review, wrote the manuscript and prepared figures. All authors reviewed and approved the manuscript.

## Conflicts of Interest

The authors declare no conflicts of interest.

## Data Availability

The data that support the findings of this study are available on request from the corresponding author. The data are not publicly available due to privacy or ethical restrictions.
